# Capture-SELEX: Selection of DNA Aptamers for Aminoglycoside Antibiotics

**DOI:** 10.1155/2012/415697

**Published:** 2012-12-30

**Authors:** Regina Stoltenburg, Nadia Nikolaus, Beate Strehlitz

**Affiliations:** Helmholtz Centre for Environmental Research (UFZ), Permoserstraße 15, 04318 Leipzig, Germany

## Abstract

Small organic molecules are challenging targets for an aptamer selection using the SELEX technology (SELEX—Systematic Evolution of Ligans by EXponential enrichment). Often they are not suitable for immobilization on solid surfaces, which is a common procedure in known aptamer selection methods. The Capture-SELEX procedure allows the selection of DNA aptamers for solute targets. A special SELEX library was constructed with the aim to immobilize this library on magnetic beads or other surfaces. For this purpose a docking sequence was incorporated into the random region of the library enabling hybridization to a complementary oligo fixed on magnetic beads. Oligonucleotides of the library which exhibit high affinity to the target and a secondary structure fitting to the target are released from the beads for binding to the target during the aptamer selection process. The oligonucleotides of these binding complexes were amplified, purified, and immobilized via the docking sequence to the magnetic beads as the starting point of the following selection round. Based on this Capture-SELEX procedure, the successful DNA aptamer selection for the aminoglycoside antibiotic kanamycin A as a small molecule target is described.

## 1. Introduction

Aptamers are a very attractive class of targeting molecules being in great demand in many fields of application, like medicine (as diagnostic and therapeutic agents) and pharmaceutics (for drug discovery and validation), as well as environmental or food analytics (as biological recognition elements). Aptamers are by nature nucleic acid molecules, but their functionality is based on their complex three-dimensional structure different from the conventional view on nucleic acids as carrier of genetic information. The complex folding of the short, single stranded DNA or RNA aptamers according to their primary sequence enables them to bind with high affinity and specificity to the given target. The intermolecular interactions between aptamer and target are characterized by a combination of complementarity in shape, stacking interactions between aromatic compounds and the nucleobases of the aptamers, electrostatic interactions between charged groups, and hydrogen bonds [1–3]. Since the first publication of aptamers in 1990 [4, 5], they have been described for a wide variety of different classes of targets from small molecules, like nucleotides, cofactors or amino acids, over peptides, polysaccharides and proteins to complex structures like whole cells, viruses and single cell organisms [6]. The growing number of aptamer publications over the years describing their selection andapplication shows the high interest in this research field. One of the challenges in this area is the optimization of the methodology to get new aptamers with outstanding binding abilities for a certain target. Aptamers are usually generated by an in vitro selection and amplification technology called SELEX—Systematic Evolution of Ligands by Exponential enrichment. [4, 5]. The SELEX process is an iterative process. From a DNA oligonucleotide library comprising a large sequence diversity and structural complexity only those oligonucleotides are selected and enriched during several SELEX rounds which can bind very tightly to the specific molecular target [7, 8]. Basic steps of the SELEX process are the binding reaction between oligonucleotides and target, washing steps to remove unbound oligonucleotides, enzymatic amplification of target-bound oligonucleotides, and purification of the selected oligonucleotide pool to subsequently start the next selection round. The best fitting sequences survive the selection procedure and represent the target-specific aptamer pool as the result of a successful SELEX process. Since the early phase of the application of the SELEX technology, it was often modified to make the selection process more efficient and less time consuming and to select aptamers with particular binding features (affinity and specificity) for different target molecules and for different applications. Variations often concern the binding conditions (buffer, temperature, and time), the efficient separation of target binding and nonbinding oligonucleotides [9], the introduction of additional selection steps, like negative or counter selection steps to remove nonspecifically binding oligonucleotides or to discriminate between closely related target molecules, the stringency of washing steps of the oligonucleotide-target complexes to improve the specificity of the aptamers to be selected, the labeling of the oligonucleotides for quantification, or the elution of the oligonucleotides from the binding complex before amplification [7]. An important aspect of an aptamer selection also is the design of the SELEX library and the use of modifications at the nucleotide level or at the ends of the oligonucleotides. Such modifications can be used to enhance the stability of the oligonucleotides necessary for the selection of RNA aptamers or to introduce new features like functional groups to provide new possibilities for the interaction with target molecules [6, 10, 11]. The numerous SELEX variants demonstrate that there is no standard selection protocol applicable to any kind of target. The selection conditions have to be carefully adapted with regard to the specific target features and the desired application of the aptamers. Medical and analytical applications for instance can have different requirements for the aptamer working conditions like buffer composition or temperature. The probe matrix often poses challenges to the aptamer stability and specificity, which can be influenced during the aptamer selection process by nucleic acid modifications and counterselection steps.

In this paper we describe the development of a SELEX variant called Capture-SELEX. It is partially derived from the FluMag-SELEX procedure published in 2005 [12], which is characterized by the immobilization of the target molecules on magnetic beads and the fluorescence labeling of the oligonucleotides for quantification. The main difference between both is the presentation of the target molecules and the oligonucleotides. The aim was to select DNA aptamers for targets which are not suitable for immobilization on solid surfaces, like small organic molecules, for example, pharmaceuticals. Therefore, a special SELEX library was developed according to Nutiu and Li, 2005 [13] to enable the immobilization of the oligonucleotides instead of the target molecules during the aptamer selection process. This paper also describes the successful application of the Capture-SELEX procedure using a mixture of pharmaceuticals (kanamycin A, sulfacarbamide, sulfamethoxazole, and sotalol hydrochloride) as aptamer selection targets. Pharmaceutical residues are found in surface, ground, and drinking water. They arise mostly from human and animal treatment. Antibiotics are an increasing issue in this context. Their introduction into the environment promotes the proliferation of genes for antibiotic resistance in bacteria. Aptamers with specificity for pharmaceuticals can be used in biosensors and assays for fast and easy detection of these substances [14]. Keeping in mind this requirement we assembled the target mixture mentioned above consisting of three antibiotics and the beta blocker sotalol hydrochloride. Using the Capture-SELEX process, an aptamer pool with specificity for kanamycin A was enriched. 

## 2. Materials and Methods

### 2.1. Chemicals

All chemicals for preparing buffers and solutions were obtained from Merck (Germany) if not mentioned otherwise. Kanamycin A disulfate salt dihydrate, sulfacarbamide, sulfamethoxazole, and sotalol hydrochloride were purchased from Sigma-Aldrich (Germany). PCR (polymerase chain reaction) components like 10×reaction buffer, 25 mMMgCl_2_, and HOTFire polymerase were purchased from Solis BioDyne (Estonia). 100 mM stock solutions of dNTPs were from GE Healthcare (Germany).

### 2.2. Capture-SELEX Library, Capture Oligo and Primers

**Figure 1:**
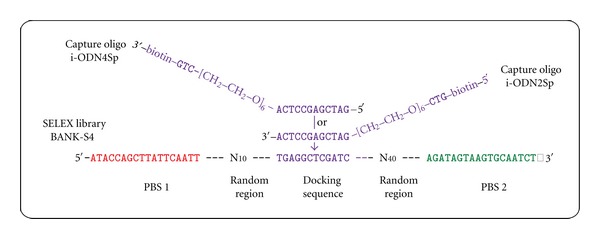
Specific composition of the Capture-SELEX library BANK-S4 and the capture oligos i-ODN2Sp and i-ODN4Sp. PBS1 and PBS2 represent the primer binding sites of each oligonucleotide in the library.

The Capture-SELEX library BANK-S4 was synthesized by Microsynth (Switzerland) including a PAGE (polyacrylamide gel electrophoresis) purification step. The library consists of a multitude of different oligonucleotides. Each of them contains specific primer binding sites (PBSs) of 18 nt at the 5′ and 3′ ends and between both two different-sized random regions (N) separated by a specific docking sequence of 12 nt: 5′-ATACCAGCTTATTCAATT—N_10_—TGAGGCTCGATC—N_40_—AGATAGTAAGTGCAATCT-3′ (Figure 1) [12, 15]. The following primers were used for amplification of the oligonucleotides during the aptamer selection process and were synthesized by biomers.net (Germany): AP10:5′-ATACCAGCTTATTCAATT-3′, AP60: the modified variant of AP10 with 5′-fluorescein, AP20:5′-AGATTGCACTTACTATCT-3′, and TER-AP20:the modified variant of AP20 with 5′-poly-dA_20_-HEGL [12]. The capture oligo i-ODN2Sp is characterized by a complementary sequence to the docking sequence of the oligonucleotides in the library: 5′-Bio-GTC-HEGL-GATCGAGCCTCA-3′ (Figure 1). It also contains three additional nucleotides and a hexaethylene glycol spacer (HEGL) at the 5′-end. The capture oligo is modified with 5′-biotin and was also synthesized by Microsynth (Switzerland) including a PAGE purification step.

### 2.3. Coupling of Capture Oligos to Streptavidin-Coated Magnetic Beads

Superparamagnetic Dynabeads M-270 Streptavidin (diameter 2.8 *μ*m) were purchased from Invitrogen/Life Technologies (USA). An appropriate amount of these streptavidin-coated magnetic beads were transferred from stock solution to a sample tube and washed three times with 500 *μ*L B&W buffer (binding and washing buffer, 10 mM Tris-HCl pH7.5, 1 mM EDTA, and 2 M NaCl). The beads were separated by placing the tube in a magnet stand. After washing the beads they were resuspended in B&W buffer to a concentration of 2×10^9^ beads/mL, and an equal volume of the biotinylated capture oligo i-ODN2Sp was added (600 pmol biotinylated oligo/1×10^8^ beads). This immobilization mixture was then incubated at room temperature for 1 h with gentle rotation using an overhead shaker (Intelli-Mixer RM-2, neoLab, Germany). The beads were separated and washed three times with 500 *μ*L B&W buffer and afterwards three times with 500 *μ*L selection buffer (100 mM NaCl, 20 mM Tris-HCl pH7.6, 2 mM MgCl_2_, 5 mM KCl, and 1 mM CaCl_2_). After resuspension in selection buffer to a final concentration of 1×10^9^ beads/mL, the streptavidin-coated magnetic beads, which are now modified with the biotinylated capture oligo i-ODN2Sp, are ready for use in the Capture-SELEX process. The bead concentration can be determined more exactly by microscopic counting (microscope Olympus BX60, Olympus Europa Holding GmbH, Germany) using a Neubauer-improved counting chamber.

### 2.4. Target Solution

Four pharmaceuticals (kanamycin A disulfate salt dihydrate, sulfacarbamide, sulfamethoxazole, and sotalol hydrochloride, see Table 1) were used as aptamer selection target mixture. Individual stock solutions of the pharmaceuticals were prepared, diluted in selection buffer, and mixed to the final concentration of 1 mM for each substance. This mixture was sterile filtered using a syringe filter with the pore size of 0.22 *μ*m (VWR, Germany), aliquoted and stored at −18°C.

### 2.5. Capture-SELEX Process

Each Capture-SELEX round starts with the thermal equilibration of the oligonucleotide pool in selection buffer. 2-3 nmol of the library oligonucleotides in the first round and, in each of the following rounds, the total quantity of selected oligonucleotides from the previous round, respectively, were heated to 90°C for 8 min, immediately cooled, and kept at 4°C for 10 min followed by a short incubation at room temperature. In parallel, an aliquot of the streptavidin-coated magnetic beads modified with the capture oligos (1×10^9^ beads in the first round and 1×10^8^ beads in each of the following rounds) was washed three times with 500 *μ*L selection buffer. The beads were resuspended in 300 *μ*L of the pretreated oligonucleotide pool and incubated overnight at 21°C with mild shaking. This step serves for the immobilization of the oligonucleotides from the pool on the magnetic beads by hybridization between the docking sequence within the oligonucleotides and the capture oligos on the beads. Unbound oligonucleotides were removed by washing the beads nine times with 500 *μ*L selection buffer. In the temperature step all of the remaining unhybridized oligonucleotides or weakened DNA duplex structures were eliminated by incubation of the DNA-bead-complexes in 500 *μ*L selection buffer at 28°C for 15 min with mild shaking and subsequently washing seven times with 500 *μ*L selection buffer. The following incubation of the DNA-bead-complexes in 300 *μ*L selection buffer at 21°C for 45 min with mild shaking serves as a control for background elution of hybridized oligonucleotides from the beads caused by the incubation procedure. After washing again seven times with 500 *μ*L selection buffer, the DNA-bead-complexes were incubated at 21°C for 45 min with mild shaking in 300 *μ*L of the target mixture (see Section 2.4). Oligonucleotides with affinity to the selection target are able to fold into a specific three-dimensional structure for binding to the target in solution and therefore are released from the DNA-bead-complexes during this target binding step. They can be collected in the supernatant by magnetic separation from the beads. 

These selected oligonucleotides bound to the target were directly amplified in 15 parallel PCR reactions. Each contained 1 *μ*M of primers AP60 and TER-AP20, 0.2 mM dNTPs each, 1.9 mM MgCl_2_, and 5 U HOTFire polymerase in PCR reaction buffer (80 mM Tris-HCl pH9.5, 20 mM (NH_4_)_2_SO_4_, and 0.02% Tween20) in a volume of 100 *μ*L. Amplification conditions were 15 min at 95°C and 30 cycles of 1 min at 95°C, 1 min at 51°C, 1 min at 72°C, and a final step of 10 min at 72°C after the last cycle. As a result, dsDNA products were obtained with a fluorescein modification at the 5′-end of the relevant sense strand and a poly-dA_20_ extension at the 5′-end of the antisense strand. Electrophoresis on 2.5% agarose gel was used to monitor the successful amplification and the correct size of the amplified DNA. An influence of the pharmaceuticals on the PCR reactions was checked and could not be observed. 

All PCR products were pooled, precipitated with ethanol in presence of linear polyacrylamide [19], and resuspended in 100 *μ*L TE buffer (10 mM Tris-HCl pH7.4, 1 mM EDTA). The two strands of the dsDNA differ in length due to the poly-dA_20_ extension of the antisense strand [20]. This was utilized for the separation of the two DNA strands in a preparative denaturing PAGE with an 8% polyacrylamide gel containing 7 mM urea and 20% formamide in TBE buffer (90 mM Tris-HCl, 90 mM boric acid, and 2 mM EDTA). The fluorescein-labeled sense strands could be identified in the gel by using a UV transilluminator. The corresponding DNA bands were cut out, and the single stranded DNA (ssDNA) was eluted from the gel with 2 mM EDTA, 300 mM sodium acetate, and pH7.8 at 80°C for 150 min with mild shaking. After removing of the gel residues by filtering through silanized glass wool, the eluted ssDNA was precipitated with ethanol in presence of linear polyacrylamide [19] and resuspended in selection buffer. A new pool of selected and fluorescein-labeled oligonucleotides was now ready for the next round of the Capture-SELEX process starting with the immobilization of the oligonucleotide pool on magnetic beads. 

The fluorescein label attached to the oligonucleotides from round two onwards enables the quantification of the oligonucleotides present in the SELEX fractions like washing steps, temperature step, background elution, and target binding. This is important in order to assess the selection progress over several SELEX rounds. By this way enrichment of specific target-binding oligonucleotides was monitored. In total, 13 rounds of this Capture-SELEX procedure were performed, and a pharmaceutical-specific aptamer pool was selected.

The selected aptamer pool from round 13 of the Capture-SELEX was amplified with the unmodified primers AP10 and AP20 and subsequently cloned into the vector pCR2.1-TOPO (TOPO TA Cloning Kit from Invitrogen/Life Technologies, USA). The resulting recombinant vectors were transformed into chemically competent *Escherichia coli* TOP10 cells (also provided by the TOPO TA Cloning Kit). Several positive transformants could be analyzed by colony PCR using a combination of a vector-specific primer (M13 forward primer or M13 reverse primer) and an aptamer-specific primer (e.g., primer AP10). This method enables a fast screening for correct plasmid inserts directly from *E. coli* colonies. The plasmid DNA of 96 clones was isolated using the QIAprep 96 Turbo Miniprep Kit from QIAgen (Germany), and the inserted aptamer DNA of each clone was sequenced (Microsynth, Switzerland). The obtained sequences were analyzed and aligned by using the web-based tool ClustalW provided by the EBI web server (http://www.ebi.ac.uk/Tools/msa/clustalw2/) [21–23]. 

### 2.6. Specificity and Affinity Tests of Aptamers

Individual aptamer candidates chosen for further characterizations were synthesized with a 5′-fluorescein label by Microsynth (Switzerland) including a PAGE purification step. Comparative binding tests with individual aptamers were performed according to the Capture-SELEX conditions using the target mixture (see Section 2.4) as well as the individual pharmaceutical solutions. Selection buffer without any target substance was used as a background control. 

Several binding tests were performed in parallel. A fresh aliquot of an appropriate amount of streptavidin-coated magnetic beads modified with capture oligos (5× 10^7^ beads for each binding test) was first washed three times with 500 *μ*L selection buffer. 50pmol aptamer per 5× 10^7^ beads was suspended in 300 *μ*L selection buffer and pretreated by heating it to 90°C for 8 min, immediately cooling to 4°C for 10 min, and keeping it at room temperature for 5-6 min before adding it to the washed beads. Incubation was performed overnight at 21°C and with mild shaking for immobilization of the aptamers to magnetic beads. On average, in each test, an amount of 31.4 ± 11.2 pmol of the aptamers (determined as the difference between the initially employed ssDNA and the ssDNA in the supernatant after the overnight immobilization step) was immobilized on (4.6 ± 0.6)×10^7^ streptavidin-coated magnetic beads modified with capture oligos. The following steps up to the background elution were identical to those described in the Capture-SELEX section. After background elution the beads were washed six times with 500 *μ*L selection buffer and evenly distributed on reaction tubes according to the number of planed binding tests. The DNA-bead-complexes were then resuspended in 300 *μ*L pharmaceutical mixture (final concentration for each pharmaceutical 1 mM), in 300 *μ*L individual pharmaceutical solutions (final concentration 1 mM) for specificity tests, in 300 *μ*L differently concentrated individual pharmaceutical solutions for affinity tests (e.g., concentration series of Kanamycin A in the range of 0–1.5 mM), or only in 300 *μ*L selection buffer as a control. Incubation was carried out at 21°C for 45 min with mild shaking. All further steps were identical to those described in the Capture-SELEX section. 

The amount of aptamer released during the target binding step from the beads was determined by fluorescence detection and calculation using a calibration curve. Fluorescence conditions were as described in Section 2.7. Solutions were centrifuged prior to pipetting them into the 96 microwell plate in order to eliminate most of the remaining beads. Aptamers used for the determination of the calibration curves were subjected to the same thermal pretreatment as the aptamers used for the specificity and affinity tests.

### 2.7. Fluorescence Detection

All fluorescence measurements of fluorescein-labeled DNA were performed on a Wallac 1420 Victor^2^ V Multilabel Counter (PerkinElmer, Germany) with excitation at 485 nm and emission at 535 nm (prompt fluorometry, time 1 s, CW-lamp energy 22500). The readings were performed in black 96microwellplates from NUNC/Thermo Fisher Scientific (Germany) with a sample volume of 100 *μ*L/well. A calibration curve in the range of 0.4–40 pmol/mL of fluorescein-labeled ssDNA prepared from the Capture-SELEX library (BANK-S4) was used to calculate the DNA concentration in samples. In the case of purchased fluorescein-labeled aptamers, a calibration curve for each of them had to be determined. No influence of the pharmaceuticals on the fluorescence readings was detected.

## 3. Results and Discussion 

### 3.1. Design of the Capture-SELEX Library and the Capture Oligos

Starting point of a selection process of target-specific aptamers is the SELEX library, comprising a huge amount of different oligonucleotides (approximately 10^15^ unique sequences), which are able to fold into distinct three-dimensional structures. This functionality is achieved through a randomized region of the oligonucleotides. A typical SELEX library consists of oligonucleotides with the same length and a central, randomized region of 20–60 nucleotides, flanked by specific sequences at the 5′- and 3′-ends. The latter serves as primer binding sites for amplification of the oligonucleotides by PCR during the aptamer selection process. This fully random library can be transformed into a partially randomized library for giving it new features, for example, by introducing defined sequences into the randomized region. Here we describe the use of such an additional, defined sequence region as docking sequence to construct a Capture-SELEX library. In a typical SELEX experiment, the target molecules are usually immobilized on a solid surface to enable an efficient separation of target-binding and nonbinding oligonucleotides in each SELEX round. This is a very crucial step for a successful aptamer selection. Conventional methods are affinity chromatography with target immobilization on different column material or the use of magnetic beads as immobilization matrix. But this methodological approach is not applicable to all potential aptamer targets, especially to very small, organic target molecules with only few or no suitable functional groups. Additional functionalization of such molecules often affects their structural features. However, retaining the native biomolecule structures of the targets is very important for the binding features of the aptamers to be selected. On the other hand, size-dependent separation techniques like filtration or centrifugation are often not practicable for small, organic target molecules. The Capture-SELEX library provides an alternative approach by immobilization of the oligonucleotides to a solid matrix instead of the targets. This is done by hybridization between the docking sequence within the oligonucleotides and a complementary capture oligo, which is coupled to the solid matrix by affinity binding or covalent binding. The selection concept is based on release of those oligonucleotides from the matrix which show an affinity to the selection target and therefore undergo a specific conformational change for binding to the target in solution. One possible risk of this concept is that not all oligonucleotides of a SELEX library are productive. Those of them which can bind to the target but do not undergo a conformational change including the docking sequence are not released and therefore are not selected, especially during the first SELEX round.

The design of the Capture-SELEX library called BANK-S4 (see Figure 1) was derived from designs described in Nutiu and Li, 2005 [13]. They firstly described an aptamer selection procedure based on the structure switching idea, with the aim to immediately transform the selected aptamers into fluorescence signaling reporter molecules for the detection of the binding complexes.

BANK-S4 is characterized by an internally unstructured 12-nucleotide docking sequence flanked by two random regions in an asymmetric arrangement. One of them was constructed relatively large with 40 nt for more structural complexity of the library. The other consists of only 10 nt. A short randomized region (20–25 nt) of a SELEX library seems to be sufficient for a successful aptamer selection, because post-SELEX optimizations like truncations often reveal a relatively short minimal functional sequence of many aptamers. However, longer random regions (at least 60–70 nt) give the libraries a higher structural complexity, for example, including high-order junctions [24] and may provide better opportunities for an interaction between the DNA molecules and the targets over an extended domain of both binding partners. The design of the docking sequence (length and nucleotide composition) has to be aimed to achieve the balance between duplex formation with the capture oligo for a stable immobilization of the library and target-dependent release of oligonucleotides from the duplex structure to form specific oligonucleotide-target complexes. The primer binding sites at the 5′- and 3′-end of the BANK-S4 oligonucleotides were derived from the FluMag-SELEX library described in Stoltenburg et al. 2005 [12].

The second part of the Capture-SELEX library concept is the capture oligo (Figure 1), which consists of 12nucleotides complementary to the docking sequence and is biotinylated for the convenient coupling to a streptavidin-modified matrix, for example, magnetic beads. Hybridization between the docking sequence and the matrix-coupled capture oligo permits the immobilization of the Capture-SELEX library. The two kinds of capture oligo design concerning the biotinylation site, at the 5′ or 3′-end of the oligos (i-ODN2Sp or i-ODN4Sp, Figure 1), implicate two alignments of the oligonucleotides contained in the SELEX library due to the asymmetric arrangement of the random sequences. Theoretically, two alignments are possible: when hybridizing to i-ODN2Sp, the longer randomized region (N_40_) is directed towards the surface of the magnetic bead, whereas when hybridizing to i-ODN4Sp, the shorter randomized region (N_10_) is directed towards the bead. The capture oligo furthermore contains a hexaethylene glycol spacer (HEGL) between the nucleotides and the biotinylation site. An alternative poly-dA_9_ spacer has proved to be less suitable for this application, because of an unwanted co-selection of T-rich oligonucleotides from the library, which are able to hybridize with the capture oligo over an extended domain including the poly-dA_9_ spacer.

The following sections describe the Capture-SELEX procedure in more detail and its successful application for selection of DNA aptamers for the aminoglycoside antibiotic kanamycin A.

### 3.2. General Procedure of the Capture-SELEX

**Figure 2:**
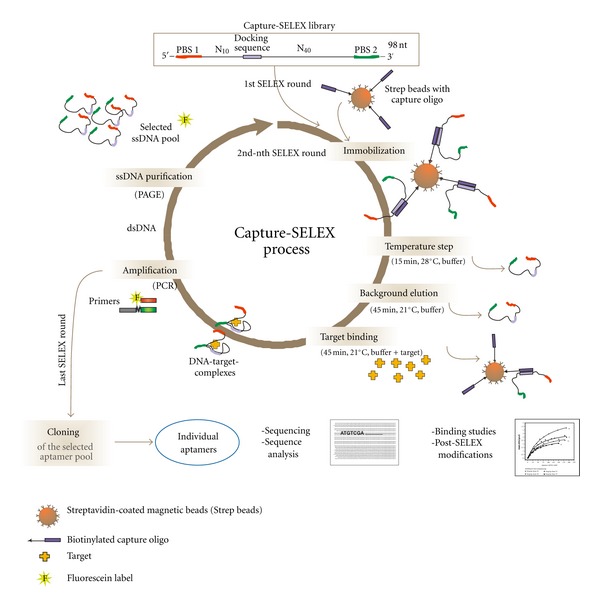
Capture-SELEX strategy.

The Capture-SELEX process using BANK-S4 as SELEX library was established and optimized as a variant of the SELEX technology for the selection of target-specific aptamers. It is characterized by capturing the oligonucleotides (SELEX library or selected oligonucleotide pool) on magnetic beads. By this way, the target molecules do not have to be immobilized and can be used in dissolved form. The magnetic separation technology offers a convenient handling and an efficient separation of bead bound components from other components of the solution [12]. The Capture-SELEX strategy is shown in Figure 2. To initiate a selection round of the Capture-SELEX process, the oligonucleotides of the library in the first round and of the selected pool in subsequent rounds, respectively, were immobilized on streptavidin-coated magnetic beads. These beads are additionally modified by coupling of the biotinylated capture oligos (Figure 1). Hybridization between both DNA partners during an overnight incubation results in the immobilization of the SELEX library or the selected oligonucleotide pool on the beads. Afterwards, the DNA-bead-complexes were incubated twice in selection buffer to reduce the unspecific release of oligonucleotides from the complexes. At first, we introduced the temperature step at an elevated temperature to eliminate unhybridized oligonucleotides or weakened DNA duplex structures. The next incubation step, the background elution step, is characterized by the same incubation conditions (time and temperature) as used for the following target binding step. We used this background elution in selection buffer to determine the amount of oligonucleotides released from the complexes caused only by the incubation procedure. Moreover, this step can easily be combined with a negative selection step by addition of appropriate substances to the selection buffer. Negative selection steps are often useful and recommended for an aptamer selection to avoid not only the enrichment of oligonucleotides that bind nonspecifically, but also to direct the selection of aptamers to a specific epitope of the target, for example, or to distinguish between closely related target molecules. After the immobilization and the different incubation steps we had to wash the DNA-bead-complexes extensively to widely remove unbound oligonucleotides. In the next target binding step the DNA-bead-complexes were exposed to the target solution. The target concentration can typically be in the range of 0.1 mM–1 mM. There is the possibility to reduce this concentration during the SELEX process for more stringent selection conditions, which can affect the affinity of the aptamers to be selected. There is also the possibility to use a target mixture (2 or more different target molecules) for a parallel selection of aptamers in one SELEX process. Oligonucleotides with affinity to the target can fold into a specific three-dimensional structure to bind to the target. These oligonucleotides are able to form a stable binding complex and are therefore released from the bead-bound state into solution during the target binding step. The remaining DNA-bead-complexes are separated, and the target-eluted DNA is directly transferred to the following amplification and purification steps of the selected oligonucleotides of one SELEX round. These steps are the same as described for the FluMag-SELEX process [12]. After the first selection round all oligonucleotides are labeled with fluorescein during amplification by PCR using a 5′-modified primer (sense primer). This enables a quantification of DNA by direct fluorescence detection and thereby the monitoring of the enrichment of target-binding oligonucleotides. We used the ratio of the fraction of oligonucleotides eluted by the target molecules (target binding step) compared to that eluted by selection buffer (background elution step) to obtain information about the aptamer selection progress in each SELEX round. A second modified primer (antisense primer) is used for PCR to introduce a nucleotide extension to the antisense strand, which permits the size-dependent separation of both strands by denaturing PAGE followed by the purification of the relevant sense strand. The resulting new pool of oligonucleotides is used for the next SELEX round starting with their immobilization on streptavidin-coated magnetic beads. Many of such Capture-SELEX rounds (typically 10–15) are necessary to enrich a target-binding oligonucleotide pool (aptamer pool). Each SELEX process ends with cloning of the PCR products of the last round to get individual aptamers. We usually choose up to 100 aptamer clones for their characterization by sequencing and sequence analysis. Binding tests with individual aptamers are then performed to screen for the best binding aptamer candidates, which are chosen for further characterization in order to get information about affinity, specificity, and minimal binding domain.

### 3.3. Selection of Aptamers for Pharmaceuticals Using the Capture-SELEX Process

In total, 13 rounds of Capture-SELEX were performed as described above (see also Section 2.5). A constant mixture of four pharmaceuticals was used as target in a concentration of 1 mM in selection buffer each (see Section 2.4.). A mixture of pharmaceuticals was chosen in order to enhance the probability to find binding sequences to at least one of the target substances as there is theoretically no hindrance of the development of aptamers to different targets during a multi-target SELEX procedure [25]. The background elution step was carried out with selection buffer without additional substances and therefore without a combination with a negative selection step. Concerning the question which one of the both possible capture oligos i-ODN2Sp and i-ODN4Sp is to prefer, the hybridization efficiencies were tested, determined as the ratio of immobilized ssDNA to the number of used beads. As the hybridization efficiencies were very similar in both assemblies, the use of i-ODN2Sp as capture oligo was decided. 

**Figure 3:**
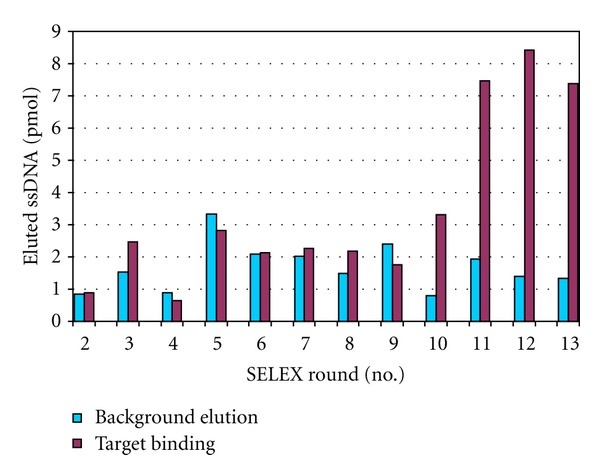
Enrichment of fluorescein-labeled aptamers during 13 rounds of Capture-SELEX for the target mixture consisting of kanamycin A disulfate salt dihydrate, sulfacarbamide, sulfamethoxazole, and sotalol hydrochloride, each 1 mM in selection buffer. Amounts of eluted single stranded DNA (ssDNA) in the target binding step (dark red) and in the background elution step (blue) are shown. As the starting oligonucleotide library initially was not fluorescein labeled, there are no bars for round 1.

In the first round, about 2 nmol of ssDNA of the SELEX library BANK-S4 was added to 7.9×10^8^ streptavidin-coated magnetic beads modified with capture oligos. An amount of 600 pmol (determined as the difference between the initially employed ssDNA and the ssDNA in the supernatant after the overnight immobilization step) was immobilized on these beads. In the following rounds, on average 45 pmol (30–70 pmol) of ssDNA from the preceding SELEX round was immobilized on 1× 10^8^ modified beads. It was determined that an amount of—on average—37 pmol (10–75 pmol) of this immobilized ssDNA remains on the beads after all washing steps as well as the temperature and background elution steps and is available for the target binding step. The concentration of the target molecules was 1 mM in 300 *μ*L each. Therefore the concentration of each target present in the sample during the target binding step was about 8,000 times higher than that of the immobilized ssDNA. Therefore the probability of oligonucleotides, once released from the capture oligos on the beads by the presence of a binding partner, to return to the capture oligos, is negligible. As can be seen in Figure 3, from round 10 on, an increase of the amount of eluted ssDNA in the target binding step hinted at the enrichment of aptamers in the process. The total amount of target eluted ssDNA increased from approximately 1 pmol to more than 8 pmol in round 12. In round 13, it decreased to 7.4 pmol and indicated that the end of the SELEX procedure was reached. The ratio of the amounts of oligonucleotides eluted in the target binding step compared to the background elution step is another parameter that can be considered for the assessment of the progress in Capture-SELEX. At first, when there is only a very small amount of specifically binding oligos in the solution, this ratio should equal unity as the conditions for target binding and background elution are the same. As a successful selection of aptamers proceeds and the amount of binders grows, the value will increase. This can be concluded from Figure 3, where the ratio of the amounts of oligonucleotides eluted in the target binding step compared to the background elution step increased from ~1 up to ~6.

**Figure 4:**
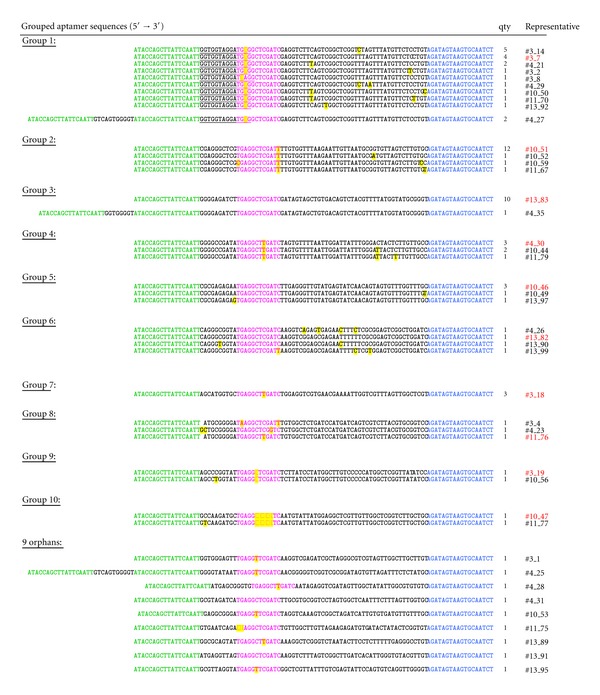
Groups and orphans of oligonucleotides (5′ → 3′) obtained after cloning and sequencing of the single stranded DNA that was selected by the target mixture (kanamycin A disulfate salt dihydrate, sulfacarbamide, sulfamethoxazole, and sotalol hydrochloride, each 1 mM in selection buffer) during 13 Capture-SELEX rounds. The representative of each group is printed in red. Each group could be divided into subgroups of totally identical sequences. The number of sequences in each subgroup is given (qty), and the representative is printed in black. Green: binding site for primer AP60 (18mer), magenta: docking sequence, originally: TGAGGCTCGATC, blue: binding site for primer AP20 (18mer), yellow marking: sites of base exchange or deletion, and underlined: possible G-quadruplex area.

After cloning and transformation of the selected aptamer pool, 99 *E. coli* clones were further examined. Of these, 96 positive transformants were determined, and the contained plasmid DNA was prepared for sequencing the inserted aptamer DNA of each clone. The 79 sequences that could be unambiguously identified [26] were grouped according to their sequence similarity into 10 groups and 9 orphans (Figure 4). The first group is the one with the largest number of members. It consists of 17 sequences that are 97 nucleotides long (one deletion occurred during the SELEX process inside the docking sequence). These 17 sequences differ from one another only in one, at the most two, base exchanges marked in yellow. In addition, in group 1 there are two sequences that are 126 nucleotides long. They obviously derived from clone #3_7 (representative of 4 identical sequences) of this group by sequence doubling of the 5′ primer binding site and the additional insert of 11 nucleotides. 

Group 2 with its 15 members (12 identical sequences and 3 sequences with single base exchanges) is much more homogeneous than group 1 as is group 3 with 10 identical clones. Additionally, there is a 124 nucleotide sequence in group 3 that again emerged from the parent sequence (clone #13_83 as representative) by doubling of the 5′ primer binding site and an additional insert of eight bases. Groups 4 (6 members), 5 (5 members), 6 (4 members), 7 and 8 (3 members each), and 9 and 10 (2 members each) are more or less homogeneous. However, no consensus sequence could be found between groups when using the web-based tool ClustalW [23].

In the sequences of group 1 an accumulation of four stretches of two to three guanine residues, respectively, separated by one to two other bases is noticeable (underlined in Figure 4). This gives the possibility for the formation of a G-quadruplex structure during the three-dimensional folding of the aptamers. 

One interesting feature of the derived sequences is the variations in the original docking sequence (TGAGGCTCGATC, printed in magenta) that are marked yellow in Figure 4. In most of the groups, changes of the original docking sequence can be found, like base exchanges, or deletions. Especially noticeable in the docking sequence of group 10, five nucleotides are lost compared to the original docking sequence.

Due to the design of the Capture-SELEX process it is necessary for potential binders to undergo conformational changes when switching from the duplex structure with the capture oligo to the specific target-binding structure. Therefore, there is a selection pressure towards docking sequences that are less stably attached to the capture oligos. On the other hand, the docking sequences have to be specific enough as hybridization has to be stable enough to survive the elution steps precedent to the target binding step. The balancing of these two demands could be observed in our selection as an increase in eluted ssDNA during the temperature elution step starting in round 3 from ~7 pmol to ~17 pmol in round 6. This amount remained high (~12 pmol) until it decreased again to ~7 pmol in rounds 12 and 13 (data not shown). Furthermore, it is not astonishing that the base exchanges in the docking sequences lead in 11 cases to G·T and in 2 cases to C·A mismatches, as these are among the most stable mismatches in DNA [28, 29], but of course, they are less strong than perfect matches. 

One representative (Figure 4, printed in red) of each of the ten groups was chosen for further examination. Those ten selected aptamers were synthesized, fluorescein tagged (Microsynth, Switzerland), and were subsequently characterized by specificity and some of them by affinity tests. 

### 3.4. Specificity and Affinity of Selected Aptamers for Kanamycin A

**Figure 5:**
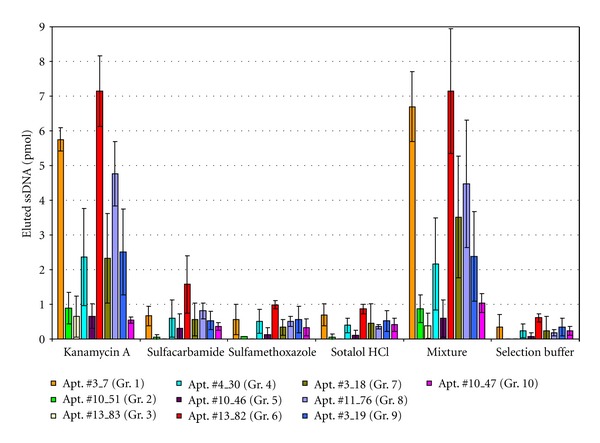
Specificity tests on the representative of each of the groups of DNA oligonucleotides derived from Capture-SELEX against the mixture of pharmaceuticals. Conditions were chosen comparably to the steps in the SELEX procedure. The four pharmaceuticals were tested individually at a concentration of 1 mM in selection buffer as well as their mixture and selection buffer alone as a negative control. The amount of eluted ssDNA was normalized to 5×10^7^ beads. Experiments were performed in duplicate or triplicate in the case of binding sequences (aptamers), the representatives of groups 1, 4, 6, 7, 8, and 9.

Firstly, the binding ability of the representatives of the ten aptamer groups to the target mixture was checked by binding tests comparable to the conditions in the selection rounds (SELEX conditions, see materials and methods section). Of the ten sequences tested, only six exhibited binding to the target mixture that exceeded the range of the negative control (as a threshold, elution of 1 pmol ssDNA was chosen to define a sequence as a binding sequence). These six sequences were the representatives of groups 1 (#3_7), 4 (#4_30), 6 (#13_82), 7 (#3_18), 8 (#11_76), and 9 (#3_19). As four possible target substances were used in parallel, it was particularly necessary to determine in specificity tests the target(s) to which the aptamers are binding. Therefore each of the four target substances was examined. In parallel, selection buffer without any target substance was tested as a negative control (Figure 5). It could be shown that most of the binding sequences were eluted only by kanamycin A (and the mixture of all four pharmaceuticals), whereas sequence #13_82 also showed weak binding to sulfacarbamide and sulfamethoxazole. Of the four pharmaceuticals, Kanamycin A is the most hydrophilic and possesses the most amino groups (see Table 1) which favor electrostatic interaction with the negatively charged phosphate backbone of the aptamer DNA and the formation of hydrogen bonds between aptamer and target. Stacking interaction of the aromatic rings of the other three pharmaceuticals with the nucleobases seems to be of minor importance here.

Besides from the binding sequences, there are four groups (groups 2, 3, 5, and 10) of oligonucleotides that do not bind to one of the four targets or the mixture. Two of them even are strong groups with many members (groups 2 and 3). The reason for this can certainly be found in the design of the Capture-SELEX. Although there are the temperature and background elution steps and a multitude of washing steps, it is still possible that some nonspecific elution during the target binding step occurs. However, the specific sequences should finally dominate the selection process. Therefore, it is especially important in the Capture-SELEX to thoroughly perform the specificity tests in order to eliminate nonbinders from the received set of sequences.

In Figure 5 standard deviations (error bars) are sometimes quite large as the experiments are very complex in its many steps, and it is therefore not easy to receive constant absolute amounts of eluted DNA. Even after the preceding centrifugation step, beads still remaining in the solution that is to be detected with fluorescence reading interfere by reflection of the excitation beam. Also the slightly different amount of beads used in each preparation, even after normalizing the amount of beads to a fixed value of 5×10^7^ beads, adds to the error. Nevertheless, this design of binding assay was chosen as it was closest to the SELEX conditions and therefore the binding reaction of the resulting aptamers to the target was expected to be unaffected by any changes in assay configuration. Furthermore, the pharmaceuticals could still be employed in solution—preferable for a future application of the developed aptamers in the detection of those pharmaceuticals in environmental samples—and without the need for immobilization and the possible risk of losing binding properties. However, in future examinations, different binding assay designs have to be tested.

**Figure 6:**
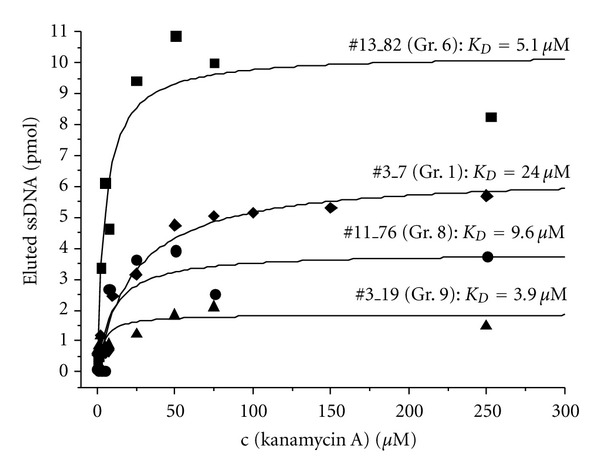
Affinity tests on the representatives of aptamer groups evolved from Capture-SELEX. Affinity tests were performed similar to the Capture-SELEX procedure, and ssDNA was eluted by different concentrations of kanamycin A in selection buffer. Data was fitted by the model of one site direct binding using a rectangular hyperbola for the saturation curve (OriginLab Corporation, OriginPro 8G SR2).

In order to determine the affinity of some selected aptamers to kanamycin A free in solution, affinity tests were performed similar to the Capture-SELEX procedure as described in the materials and methods section. Upon target binding of the selected aptamer to kanamycin A in different concentrations, certain amounts of fluorescein-labeled aptamer are released from the streptavidin-coated magnetic beads modified with capture oligos. These amounts are then quantified by fluorescence detection (Figure 6), and the resulting data is fitted by the model of one-site direct binding using a rectangular hyperbola also known as binding isotherm or saturation binding curve (OriginLab Corproration, OriginPro 8G SR2). The equation used for this model describes the equilibrium binding of a ligand to a receptor as a function of increasing ligand concentration, and *K*_*D*_ is the equilibrium dissociation constant. When the target concentration equals *K*_*D*_, half of the binding sites (aptamers) are occupied at equilibrium. The derived dissociation constants, *K*_*D*_, for aptamer representatives of the groups 1, 6, 8, and 9 are in the low micromolar range ((#3_7 (gr. 1): 24 *μ*M, #13_82 (gr. 6): 5.1 *μ*M, #11_76 (gr. 8): 9.6 *μ*M, and #3_19 (gr. 9): 3.9 *μ*M). 

Using a different SELEX method (affinity chromatography with kanamycin-immobilized sepharose beads), Song et al., 2011 only recently selected DNA aptamers for kanamycin [27]. They started with a synthetic ssDNA library of 90 nt in length with a random sequence of 40 nt flanked by two primer binding sites. Kanamycin was immobilized on CNBr-activated sepharose beads. These beads were used for the preparation of an affinity column for kanamycin binding oligonucleotides. After nine rounds of selection, they obtained 16 individual sequences out of 48 clones. A T-GG-A motif with a stem-loop folding pattern was present in 11 of the sequences and was assumed to be the binding region of the aptamers. The affinity of aptamer Kana2 to kanamycin immobilized at magnetic beads was tested, and a *K*_*D*_ value of 85.6 nM is stated. 

**Figure 7:**
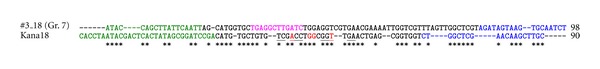
ClustalW [23] comparison of the sequences for kanamycin binding aptamers derived from different types of SELEX. #3_18 (gr. 7): aptamer developed by Capture-SELEX as described here. Kana18: aptamer developed by Song et al., 2011 [27] using affinity chromatography with kanamycin-immobilized sepharose beads. Fixed regions are marked with green (5′-primer binding regions), blue (3′-primer binding regions), and magenta (docking sequence). Random regions are printed in black. Red: binding region as assumed by Song et al., 2011 [27] and underlined: stem-loop forming sequence as given in [27].

In order to find possible sequence similarities, we checked the sequences of the representatives of the ten groups of oligonucleotides that we found in our Capture-SELEX against all those 13 sequences shown in the publication of Song et al. [27], using the web-based tool ClustalW [23]. We compared the regions that were given beforehand (the primer binding sequences and in our case the docking sequence, resp.) and the random regions. The highest similarity—especially in the random regions—showed one pair of sequences (our sequence #3_18 (gr.7) and their Kana18). However, no consensus was found between the sequence that was determined as binding region by Song et al. and our sequences (Figure 7). Therefore, a different binding motif has to be assumed in our case. The experiments in order to find the binding region within our selected aptamers will follow. 

### 3.5. Aptamer Assay Development Based on Capture-SELEX Principle

Analytical applications of aptamers often require some post-SELEX modifications. Typical modifications are the attachment of functional groups, for example, for immobilization of the aptamers on sensor surfaces, the attachment of reporter molecules for monitoring the aptamer-target binding, sequence alterations, or a combination of them. Different strategies have been developed to transduce aptamer-target interactions into a recordable signal [30–33]. However, the risks of such modifications are the possible loss of the affinity and specificity of the aptamers for their targets. To circumvent this problem, a few alternative methods for a direct selection of fluorescence-signaling aptamers have been described [13, 34–36]. These *in vitro* selection processes are characterized by the inclusion of signaling components like reporter molecules for fluorescence-based detection, special sequences, or additional oligonucleotides, so that large post-SELEX modifications can be avoided. 

**Figure 8:**
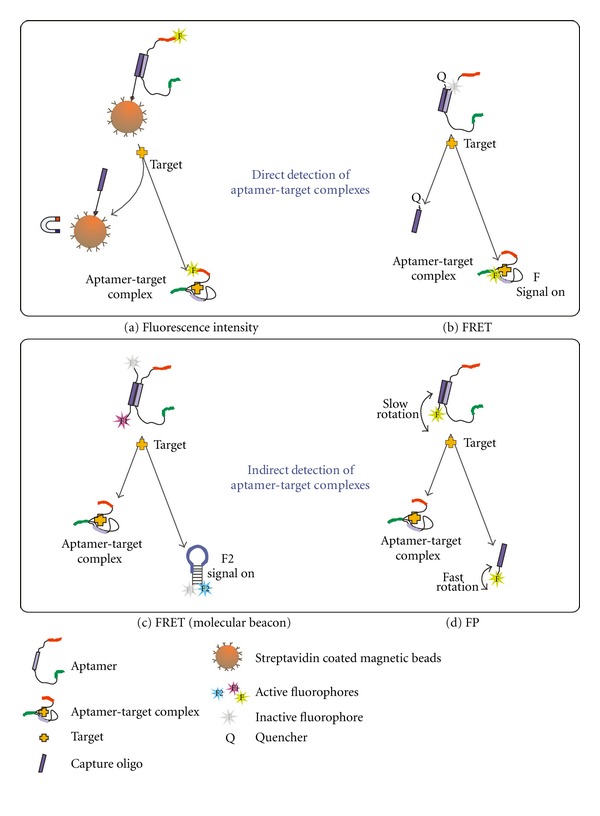
Potential assay strategies using aptamers selected by the Capture-SELEX principle. The switch from the hybridized to the dehybridized stage of the aptamers after target addition can be used to generate a recordable signal based on fluorometric methods like changes in fluorescence intensities, fluorescence resonance energy transfer (FRET), FRET with molecular beacon formation, or fluorescence polarization (FP).

The Capture-SELEX principle, described in this paper, also offers the possibility to develop detection assays based on the duplex formation between the docking sequence of the selected aptamer and the capture oligo. The target-dependent conformational change leads to the release of the aptamer from the duplex structure with the capture oligo. This switch from the hybridized to the dehybridized stage of the aptamer can be used to generate a recordable signal.  Figure 8 shows potential strategies for an assay development based on fluorometry using well-known methods like changes in fluorescence intensity, fluorescence resonance energy transfer (FRET), molecular beacon, or fluorescence polarization (FP). The strategies shown in Figures 8(a) and 8(b) enable the direct detection of aptamer-target complexes. The aptamer is labeled with a fluorophore and therefore the binding complex formed after addition of the target can be measured directly by fluorescence detection. The strategies in Figures 8(c) and 8(d) are characterized by indirect detections of aptamer-target complexes. This means the aptamer is unlabeled, whereas the capture oligo is labeled with one or two fluorophores. After addition of the target the aptamer is released from the duplex structure with the capture oligo causing changes in the fluorescence behavior of the capture oligo, which can be detected instead of the aptamer-target complex.  Figure 8(a) represents the same strategy as used during the Capture-SELEX process. We have applied this assay strategy for screening for the best binding aptamer candidates after cloning and sequencing of the selected aptamer pool and also for specificity and affinity tests (see Section 3.4.). All other assay strategies are based on different modifications mostly of the capture oligos (attaching of fluorescence reporter molecules and sequence alterations). Figures 8(c) and 8(d) show strategies for the detection of aptamer-target complexes without any modifications of the aptamers. Our future work aims at the development of an aptamer assay for kanamycin A using the described strategies.

## 4. Conclusions

A special variant of the SELEX technology, the Capture-SELEX process, has been established for the selection of DNA aptamers for those targets, which cannot be immobilized on a solid surface, for example, small organic target molecules. But the immobilization of one of the binding partners is a very important and convenient method to enable an efficient separation of target-binding and nonbinding oligonucleotides during the aptamer selection procedure. Therefore, a partially randomized DNA library with a defined docking sequence within the random region has been designed to hybridize with capture oligos on magnetic beads. By this way, the oligonucleotides of the library can be immobilized instead of the target molecules. An extensive washing of the resulting DNA-bead-complexes is recommended to remove all unhybridized DNA molecules. Moreover, two special elution steps are used prior to the target binding step to minimize the amount of oligonucleotides that is released nonspecifically from the beads, for example, caused by incorrect duplex formation or by mechanical forces. For the assessment of the progress of selection in each SELEX round, the direct comparison between the background elution step and the target binding step is used. The amount of oligonucleotides eluted in presence of the target should significantly exceed the background elution and should further increase (until reaching a maximum level) during the Capture-SELEX process. A fluorescein label serves for the quantification of the DNA in the different SELEX fractions of each SELEX round (from the second round on) by fluorescence detection. 

The configuration of the Capture-SELEX process is very flexible. The background elution step can easily be combined with a negative or counterselection step in order to influence the specificity of the aptamers to be selected. In addition to single target substances, the Capture-SELEX process is applicable to target mixtures containing two or more different substances for a parallel selection of differently targeted aptamers. Moreover, the Capture-SELEX principle is equally attractive for other classes of target molecules, like polysaccharides, peptides, or proteins.

The successful application of the Capture-SELEX process for the selection of DNA aptamers for kanamycinA was demonstrated, and dissociation constants were determined. For a more precise examination of the received aptamers, binding specificities towards other aminoglycoside antibiotics have to be determined. This is under investigation and will be subject of a following publication.

The Capture-SELEX principle also provides the possibility to develop fluorescence-based detection assays without extensive additional modifications of the selected aptamers. The switch of the oligonucleotides between the duplex structure (with the capture oligos) and the aptamer-target complex upon addition of target molecules can be used to generate a measurable signal. Therefore, future work will aim at the assay development for a sensitive detection of kanamycin A based on the particular structural features of the selected aptamers, as depicted in Section 3.5.

## Figures and Tables

**Table 1:** Characteristics of the four pharmaceuticals used in the Capture-SELEX process as target mixture.

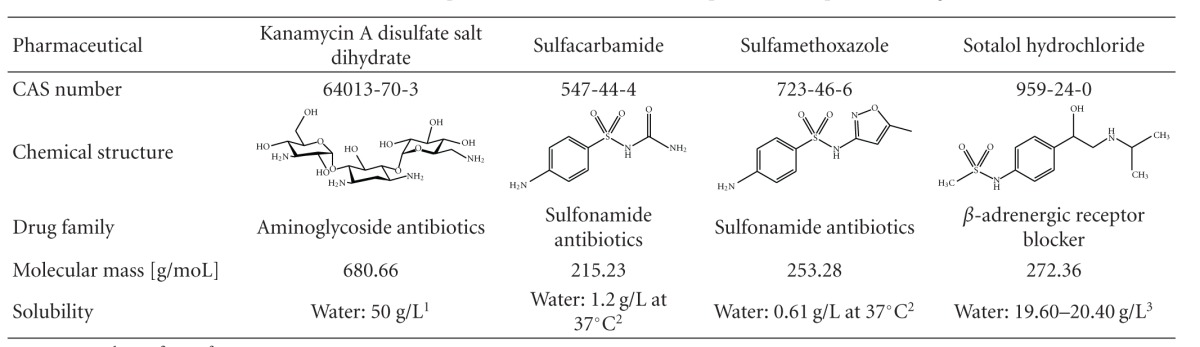

Data sources: ^1^[16], ^2^[17], ^3^[18].
